# Translation of the International Physical Activity Questionnaire (IPAQ) in Various Indian Languages: A Narrative Review

**DOI:** 10.7759/cureus.112567

**Published:** 2026-07-13

**Authors:** Noel S Macwan, Sarvang M Desai

**Affiliations:** 1 Physical Medicine and Rehabilitation, Sumandeep Vidyapeeth (Deemed to Be University), Vadodara, IND; 2 Orthopaedics, Smt. B. K. Shah Medical Institute and Research Centre, Sumandeep Vidyapeeth (Deemed to Be University), Vadodara, IND

**Keywords:** indian population, international physical activity questionnaire, ipaq, measurement, physical activity, reliability, translation

## Abstract

One of the numerous issues India faces as a developing country is the shift in public health and nutrition. The nation’s health is at risk due to physical inactivity, which is a separate risk factor for obesity and several other chronic illnesses. Physical activity (PA) is one of the most important habitual behaviors that support a healthy life by preventing illnesses and increasing health benefits. Maintaining and improving health requires regular PA. Numerous methods are available for measuring PA. To collect PA estimates that may be compared between different populations or across national borders, the International Physical Activity Questionnaire (IPAQ) was developed and is utilized worldwide. The use of the IPAQ, which is translated into Indian languages, is the main topic of this review. Databases such as PubMed, Scopus, Semantic Scholar, and Google Scholar were used to conduct a thorough literature search in March 2026 by two independent researchers. This review focused on research on the translation of the IPAQ into Indian languages. The keywords used for the search strategy were combined using the Boolean terms “Physical activity” OR “Physical inactivity” AND “Measurement” OR “Assessment” AND “Indian Language” AND “Reliability” AND “IPAQ” AND “Translation.” The initial search identified 13 studies based on the title and abstract. After removing the five duplicate studies and three studies with abstract only, five studies meeting the inclusion criteria were reviewed. Although electronic instruments would be more reliable, they are not suitable for epidemiological studies due to India’s large population and economic constraints. For the purpose of evaluating PA, translation of the IPAQ into different local Indian languages is the need of the time.

## Introduction and background

Any skeletal muscle-driven movement of the body that necessitates the use of energy is referred to as physical activity (PA) [1-3]. PA can be divided into distinct areas, including transportation, domestic duties, leisure time PA, and vocational PA [3]. PA is one of the most significant habitual behaviors that promotes a healthy life by preventing illnesses and boosting health benefits [4]. Regular PA is crucial for maintaining and promoting health [5,6].

Because it can lower the risk of chronic illness, early death, functional limits, and disability, regular PA is crucial for good aging [7]. PA levels have been falling in many nations, despite the well-established health advantages of PA in reducing type 2 diabetes, cardiovascular illnesses, hypertension, various malignancies, and deteriorated psychological health [2,6,8]. Exercise has been shown to either completely remove the risk or reduce the likelihood of developing such serious diseases among the young members of society who will shape the future [9]. As a result, PA offers a wide range of health-related advantages, such as decreased risk for several illnesses and enhanced functional capacity [6].

The World Health Organization (WHO) advises engaging in PA as per the following protocol: “at least 150 minutes of moderate-intensity aerobic PA or 75 minutes of vigorous-intensity aerobic PA per week, or an equal combination of both, to enhance fitness and endurance levels and lower the risk of non-communicable illnesses” [2,10]. For the Indian population, there has been an effort to propose a few changes in PA levels recommended by the WHO [11].

The cost-effectiveness of self-report surveillance in tracking large, diversified populations in both urban and rural environments makes it essential for India. It is the sole feasible method for capturing the “context” of activities that comprise the great majority of PA in the country, such as occupational labor and active transportation [12]. Moreover, public health and nutritional transition are among the many challenges India faces as a developing nation. Indians are shifting from traditional food rich in cereals and fibers to fast food high in sugar, lipids, and protein-rich animal foods (ready-to-eat food culture), which are linked to various non-infectious diseases observed late in life [9]. Physical inactivity is a major risk factor for osteoporosis, type 2 diabetes, cardiovascular disease, and several forms of cancer [13]. It is also known that sedentary lifestyles and being bedridden or homebound are particularly dangerous for older individuals. Physical inactivity is an independent risk factor for obesity and several chronic diseases and ailments that endanger the population’s health [3].

The International Physical Activity Questionnaire (IPAQ) was developed to offer a collection of well-prepared tools that can be applied globally for gathering PA estimations that can be used for comparison between various inhabitants or across national borders [8,14-16]. The IPAQ is one of the most popular PA questionnaires for adults [14]. It is a self-administered questionnaire with acceptable validity and reliability for assessing PA levels and patterns in healthy individuals between the ages of 15 and 69 years. IPAQ comes in two forms, i.e., the long-form and short-form, with reference periods of “the usual week” or “the last seven days.” Seven short-form questions and 27 long-form questions are included [2]. Accurately measuring activity levels through different domains of PA (transportation, leisure time, occupational, household, etc.) among the inhabitants under study is crucial for describing, monitoring, and potentially implementing effective interventions for PA [8].

The IPAQ has been translated into numerous languages, cross-culturally modified, and evaluated in several nations worldwide [2]. India is a pertinent option to assess the IPAQ for cultural and psychometric relevance because it is the most populous country on the Asian continent and has varied cultures, varied ethnicities, and multilingual inceptions similar to those of other Asian nations [6].

To our knowledge, there have not been any reviews that have studied all the translated versions of the IPAQ in different Indian languages. Therefore, this review aims to focus on studies that have translated the IPAQ scale into different Indian languages.

## Review

Methodology

For studies to be included in this review, they needed to meet the following criteria: studies addressing the translation of any type of IPAQ into different Indian languages, studies published after 2010, and studies published in the English language. Exclusion criteria for the review were studies that had IPAQ translated into languages other than Indian languages.

A detailed literature search was conducted in March 2026 using databases including PubMed, Scopus, Semantic Scholar, and Google Scholar by two independent researchers (NM, SD). This review examined studies relevant to the IPAQ in the Indian population using the translated version of the IPAQ in the Indian languages. The keywords used for the search strategy were combined using the Boolean terms “Physical activity” OR “Physical inactivity” AND “Measurement” OR “Assessment” AND “Indian Language” AND “Reliability” AND “IPAQ” AND “Translation.” Data were extracted from each study focusing on the IPAQ scale translated into different Indian languages.

Results

**Figure 1:**
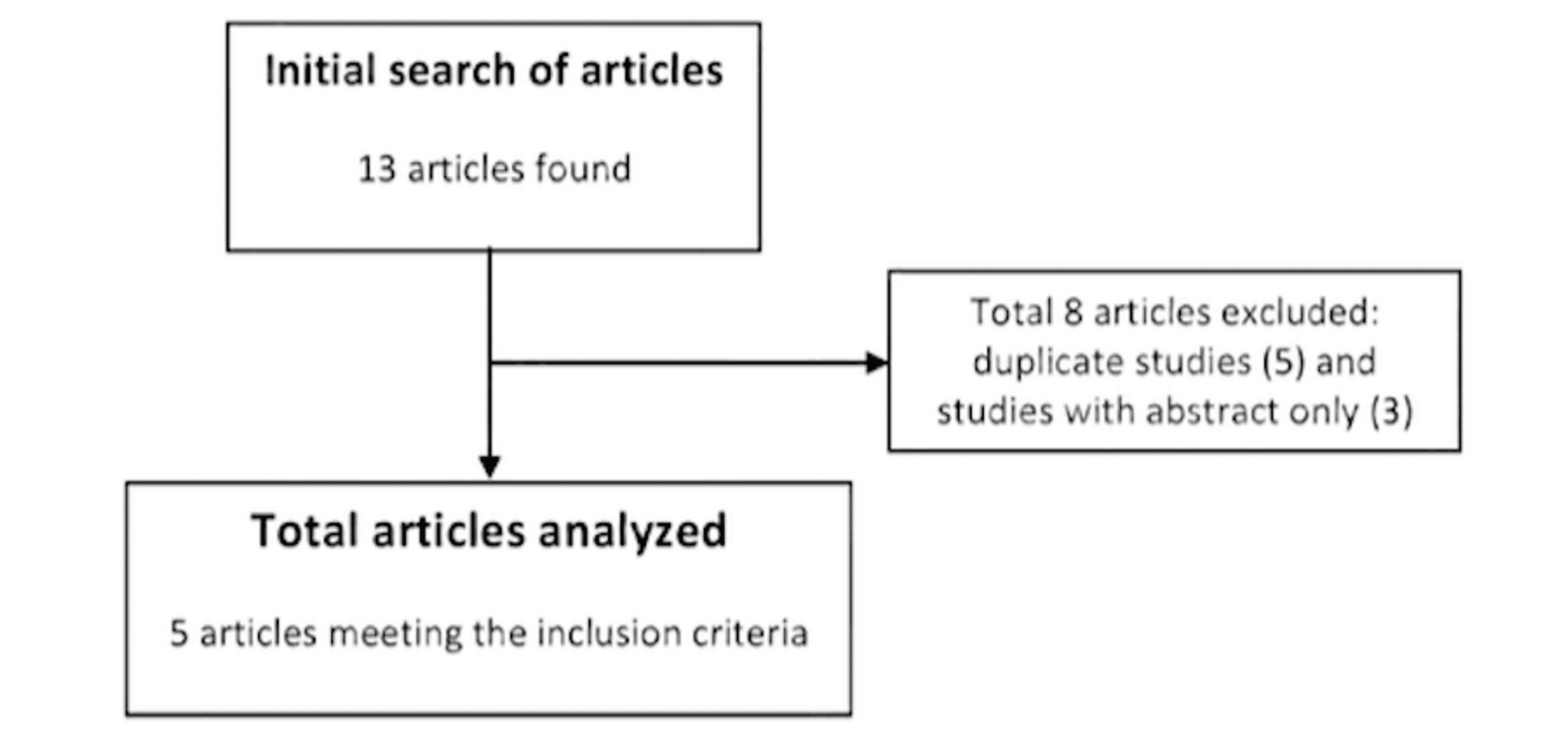
Flowchart illustrating study selection.

The initial search identified 13 studies based on title and abstract screening. After removing the five duplicate studies and three studies with abstract only, five studies meeting the inclusion criteria were reviewed (Figure 1).

Apart from the English version of the IPAQ, which is used globally, five articles showed the use of five different languages, including Hindi, Urdu, Marathi, Gujarati, and Punjabi, into which the IPAQ has been translated. A summary of IPAQ scales translated into the Indian languages is presented in Table 1.

**Table 1:** A summary of studies detailing the translation of the IPAQ in different Indian languages. IPAQ = International Physical Activity Questionnaire; IPAQ-LF = long-form International Physical Activity Questionnaire; IPAQ-SF = short-form International Physical Activity Questionnaire; InS = Indian subcontinent; PA = physical activity; ICC = intraclass correlation coefficient

Study	Translation language	Population	Methodology	Cronbach’s alpha	Test-retest reliability (ICC)	Corelation coefficient	Conclusion
Prabhu and Thakur [2]	Hindi	60 participants for reliability and validity analysis within the age group of 20–69 years were randomly selected	The Hindi IPAQ-LF was administered to 60 individuals twice over the course of two weeks to evaluate the test-retest reliability. A seven-day pedometer recording was used to compare the construct validity	0.82	0.963	0.783	The IPAQ-LF in Hindi is a valid and trustworthy measure for determining the degree of PA among Hindi-speaking people
Wani and Nabi [6]	Urdu	198 participants (50% women) with a mean age of 35.6 years (SD = 10.3)	A cross-sectional study to assess the InS IPAQ-LF’s validity and reliability in relation to several biological factors was conducted. A total of 198 individuals were chosen from neighborhoods with varying PA and socioeconomic conditions	Not specified	0.79	Not specified	The InS IPAQ-LF showed substantial evidence of test-retest reliability and might be useful for assessing adults’ context-specific PA mannerisms
Bote and Mahajan [1]	Marathi	100 healthy individuals between the age group of 18–65 years from Dr. A.P.J. Abdul Kalam College of Physiotherapy, PIMS, Loni were recruited using simple random sampling	The inclusion and exclusion criteria were used to screen the participants. The IPAQ Marathi was administered to the participants. The responses were converted to weekly metabolic equivalent task minutes. The uniaxial accelerometer watch was used for the validation	0.7	0.43	0.65	For the measurement of PA among Maharashtra’s rural people, the translated IPAQ version demonstrated adequate validity and reliability
Rathod et al. [17]	Gujarati	A cross-sectional study was conducted at the Government Physiotherapy College, Jamnagar, Gujarat, India. For face and content validity, participants were experts (n = 5) in the fields of medicine, physiology, and cardiopulmonary physiotherapy	The forward-backward-forward method was used to translate the IPAQ from English into Gujarati. The Gujarati version’s face and content validity were assessed using the group consensus method. A team of specialists in the fields of medicine, physiology, and cardiopulmonary physiotherapy reviewed each question. Each question was analyzed for content, meaning, wording, format, ease of administration, and scoring. Each question was scored by an expert group as accepted, rejected, or accepted with modification. The procedure was continued until 80% consensus was reached for all items. Concurrent validity was found by comparing the English version of the IPAQ with the Gujarati version of the IPAQ	Not specified	Not specified	0.9	The IPAQ’s Gujarati version is valid for usage among Gujarati people
Shenoy et al. [18]	Punjabi	A cross-sectional study was conducted in Amritsar among 100 apparently healthy adults.	An expert committee reviewed and pretested the English IPAQ-SF after it had been translated into Punjabi, synthesized, and back-translated. The final questionnaire (Punjabi IPAQ-SF) was tested for concurrent and construct validity	Not specified	Not specified	0.994–1	For the measurement of PA in India, the Punjabi IPAQ-SF showed good concurrent validity across all the variables

The quality analysis of the included studies was performed using the scoring system used for evaluating a study other than a clinical trial [19] by the same independent researchers (NM and SD) who conducted the extensive literature search. Every article was screened for eight different criteria, including study purpose, design, subjects (inclusion and exclusion criteria), method/data collection, outcome measure, statistical analysis, authors’ conclusion, and clinical impact. Both independent researchers graded all five included articles on a 10-point, 5-point, or 0-point scale based on the availability of the particular criteria in the study, other than the last criterion for clinical impact. The last criterion, clinical impact, was graded as 30 points, 15 points, or 0 points by both independent researchers [19]. The total score was calculated for every study out of 100 points by both researchers and later finalized for every study. Any discrepancy in the scores by both individual researchers was resolved by mutual understanding. A study scoring 80 or more was considered to be of good quality. The final quality grading of the included studies is presented in Table 2.

**Table 2:** Quality of the included studies (non-randomized clinical trial in origin).

Study	Score for the non-clinical trials (out of 100)	Study quality
Prabhu and Thakur [2]	95	Good
Wani and Nabi [6]	90	Good
Bote and Mahajan [1]	90	Good
Rathod et al. [17]	85	Good
Shenoy et al. [18]	90	Good

Discussion

As mentioned earlier, the present review took into consideration different articles that discussed the IPAQ scale translated into different Indian languages. There are many different languages spoken in India. The real number of languages is estimated differently. Overall, 462 distinct languages are listed in Ethnologue (accessed on September 10, 2018), of which 448 are living, and 14 are extinct. Despite being members of several language families, the languages of India (the Indian subcontinent) have common linguistic characteristics. India has therefore been referred to as a linguistic region [20].

As Hindi is the primary and official language of India, Hindi speakers make up about 41% of India’s overall population [2]. The Marathi language has been culturally altered to fit Indian customs and ways of life, particularly in Maharashtra’s rural districts. The majority of people who live in Maharashtra’s rural districts speak Marathi daily [1], whereas the Gujarati language is mostly preferred by people residing in Gujarat [17]. With 3% of Indians speaking it, Punjabi is the 10th most spoken language in India [18]. The Indian states of Himachal Pradesh, Punjab, and Haryana have made it their official language. Additionally, it is the third most widely spoken language on the Indian subcontinent. Punjabi is the 10th most widely spoken language in the world, with 130 million native speakers [18].

PA can be measured using a variety of techniques. These include objective techniques such as accelerometers, pedometers, and heart rate monitors; subjective techniques such as structured questionnaires and activity logs; and criteria techniques such as doubly labelled water, indirect calorimetry, and direct observation. However, due to its lower price, convenience for usage, and comparative simplicity in measuring the energy utilized, questionnaires have typically been the instrument employed in large-scale epidemiological research [8]. Although self-report techniques for measuring PA and sedentary behavior, including logs, questionnaires, or surveys, are frequently simple to use, they cannot be entirely trustworthy due to recollection bias and mistakes. Due to their affordability, PA surveys are frequently the technique of choice for assessing PA [3].

In an effort to standardize the assessment of PA prevalence across many nations and cultures worldwide, the IPAQ was created. In 2000, eight distinct IPAQ forms were created, and their validity and reliability were examined in 14 locations across 12 nations [21]. The IPAQ is available in two versions (the long form and the short form). There are two types for each version: telephonic or in-person interviews and self-administered [1,17]. The short IPAQ is a useful tool for estimating general moderate-to-vigorous PA levels; it was created as a population surveillance tool and is unable to obtain domain-specific PA data (e.g., discriminate between transport-related and leisure activities) [15]. Globally, the long IPAQ has proven to be an effective tool for evaluating adult PA. Additionally, it is being utilized in global programs that examine how environmental factors affect adult PA [22].

Men and women take on a great deal of responsibility in their personal and professional lives during middle age. Their priorities shift from themselves to their families, careers, and other obligations. According to a number of Indian researchers, sedentary behavior grows increasingly prevalent as people age. Compared to their younger peers, they have a larger possibility of gaining weight and developing chronic illnesses [23].

Illness prevention is thought to be the best course of action, particularly in developing and underdeveloped nations where the majority of people cannot afford secondary or tertiary healthcare due to the high illness load. Numerous studies have demonstrated that increasing PA increases psychological wellness and lowers the risk of cardiovascular disease, type 2 diabetes, and stroke. Promoting physical exercise is crucial for lowering the prevalence of non-communicable diseases [1].

Measuring PA behavior is helpful for a variety of purposes, including tracking secular behavioral patterns and assessing the success of programs and treatments, in addition to understanding the relationship between PA and health [1]. Other behaviors mentioned in PA guidelines (muscle strengthening, balancing activities (for older individuals aged ≥65 years), and inactive time) are not consistently and adequately monitored to generate global estimates and targets [24,25].

Adults in India were given a modified version of the short-form IPAQ, and the results showed strong support for test-retest reliability, which is consistent with some other research. However, it is crucial to assess the long-form IPAQ for applicability in India, as the short-form IPAQ does not have domains and does not offer domain-specific details on PA behavior in a particular context [6].

As the primary and official language of India, Hindi is extensively spoken in the country. However, currently, there is no Hindi version of this questionnaire’s lengthy form. Therefore, the IPAQ must be translated, culturally adjusted, and appropriately assessed for psychometric qualities to be used for investigation among non-English-speaking communities in India. As India’s sociocultural and physical environments are different from those of other countries, just translating the IPAQ might not be enough to preserve its content validity. Therefore, a cross-cultural modification of the questionnaire is required to preserve the conceptual equivalency [2].

Test-retest and internal consistency reliability were assessed for the Hindi version of the long-form IPAQ. With an intraclass correlation coefficient (ICC) ranging from 0.83 to 0.96, the total PA, employment, transportation, housekeeping, and leisure domains all had strong reliability. The transportation sector had the lowest dependability (0.83), while the leisure sector had the highest reliability (0.93). The long-form IPAQ in the Hindi version, which may be utilized to gauge PA levels among people speaking the Hindi language, has good to outstanding reliability and strong validity [2].

To represent the realities in the Indian subcontinent, several cultural changes were made to the original questionnaire [3]. Additionally, the questionnaire was independently translated from English into Urdu after adaptation. The 31 questions of the Indian subcontinent version of the long-form Indian subcontinent IPAQ, which is accessible in Urdu, enquire about PA for the previous seven days in the form of PA frequency (days/week) and duration (minutes/day). The modified long-form IPAQ items had moderate construct validity and acceptable test-retest reliability among adults in the Indian subcontinent. With comparatively greater associations among the test-retest for complete PA, work-related PA, sitting, and strong-intensity activity, they discovered indications of good reliability. The ICC values for PA domains remained above 0.90, an extent of consistency that has been deemed acceptable for IPAQ scores, except for walking intensity PA and active transport [6].

The IPAQ has been translated from English into the Marathi language using the round-trip translation method, following the guidelines provided in the IPAQ manual for validity and reliability. Following the instructions provided by the corresponding authors, the questionnaire was translated into Marathi. In addition to using the Misfit accelerometer watch for a week, the subjects were required to complete the questionnaire, namely, the short-form IPAQ-M [1]. There was a moderately significant link between the total time spent and the IPAQ score, with Pearson’s correlation coefficient (r) of 0.65, and the reliability was also shown to be satisfactory with Cronbach’s alpha (r) of 0.70. The findings showed that the short-form IPAQ-M had acceptable qualities to assess PA in people with good health [1].

The Gujarati and English versions of the IPAQ have comparable validity indices. For evaluating PA in healthy persons, the Gujarati version of the IPAQ has adequate qualities. The panel of experts did not reject any of the questions for face and substance validity. Overall, 21 questions translated into Gujarati were approved. Only six questions needed expert discussion before they could be modified. In questions 8 and 9, two-wheeled vehicles were utilized instead of trams. In Gujarat, two-wheelers are a more popular form of transportation than trams. As snow shovelling is not feasible given Gujarat’s location, question number 14 was not retained as an example. Other than double tennis, various other sports are more popular in Gujarat; the words “other sports” were added to question number 24. Six weekdays, or Monday through Saturday, were taken into consideration in question number 26 because it is the Gujarati system. In question 27, even just one day (Sunday) was taken into account for the weekend [17].

Apart from long-form IPAQ, medium IPAQ, and short-form IPAQ, IPAQ elderly assesses the PA levels among the elderly. As per other IPAQ scales, IPAQ elderly also consists of five domains and measures the level of low-to-vigorous activity performed by the elderly. It analyzes the total time spent on different activities across different domains [26].

Strengths of the study

The present review took into consideration the SANRA (Scale for the Assessment of Narrative Review Articles) scale, modified in 2014, for the analysis of narrative reviews. The six items in the scale analyze the construct of the narrative review, thereby improving the quality of the review [27,28]. The standard of the five studies was appraised by using the scoring system for evaluating a study other than a clinical trial [19].

Limitations of the study

The reliability of structural differences of the long-form IPAQ and short-form IPAQ was not critically contrasted to determine their impact in the Indian context. Even though the present study’s quality was kept into consideration, the present study did not take into consideration the ongoing studies that were in the process of translation into other different Indian languages.

Clinical implications

The present study reviews different studies focusing on the translation of the IPAQ into different Indian languages. India, being a multilingual country with the second-largest population in the world, needs to monitor the PA levels of the population for the healthy development of individuals. Availability of the IPAQ in different local Indian languages will help to reach the remote places of the country to monitor the PA levels of individuals by administering the IPAQ scale in their own native language. This will help gather more epidemiological data related to PA, thereby enabling planning to improve and maintain the PA levels with appropriate measures.

## Conclusions

Finding a cohesive method to coordinate different approaches is a constant challenge for health strategies. Electronic devices would undoubtedly be more dependable, but given India’s large population and financial limitations, they are not appropriate for epidemiological research. Therefore, it is necessary to get a consensus on a viable and trustworthy tool such as IPAQ for assessing PA levels in India, where various languages are spoken natively. The IPAQ scale has been translated into five different Indian languages so far. The scale needs to be further translated into other Indian languages so it can be administered to a population speaking varied local languages other than the languages in which it has already been translated.
